# Evaluation of X-ray doses and their corresponding biological effects on experimental animals in cone-beam micro-CT scans (R-mCT2)

**DOI:** 10.1007/s12194-015-0334-1

**Published:** 2015-10-06

**Authors:** Nobuyuki Miyahara, Toshiaki Kokubo, Yukihiro Hara, Ayuta Yamada, Takafumi Koike, Yoshinori Arai

**Affiliations:** National Institute of Radiological Sciences, 4-9-1 Anagawa, Inage-ku, Chiba, 263-8555 Japan; Rigaku Co., 3-9-12 Matubara-cho, Akisima-Shi, Tokyo, 196-8666 Japan; Nihon University School of Dentistry, 1-8-13 Surugadai Kanda, Chiyoda-ku, Tokyo, 101-8310 Japan

**Keywords:** Micro-CT, Radiation damage on mice

## Abstract

Studies show that the radiation dose received during a micro-CT examination may have adverse effects on living subjects. However, the correlations between the biological effects and the radiation doses have never been thoroughly evaluated in the majority of cases. In this study, we evaluated the biological radiation effects of measured radiation doses in ICR mice using cone-beam micro-CT scans. Long-term in vivo whole-body micro-CT scans of ICR mice were performed for a duration of 4 weeks. Although a scanning frequency of three scans per week is higher than that necessary for conventional studies, this study represents particular cases where the subjects may undergo an extreme number of examinations. The average X-ray dose of a CT scan measures 16.19 mGy at the center of a phantom and 16.24 mGy at an offset position of 7.5 mm from the center of the phantom. The total average dose at the center of the phantom during the 4-week scanning period was 194.3 mGy. No significant radiation effects were observed in the weight gain curves, organ weights, blood analyses, litter sizes, reared offspring sizes, and the histopathologic results. Therefore, it is unlikely that the measured doses for the CT scans caused any radiation damage in the mice.

## Introduction

The development of various animal disease models and the clinical simulations of pathologic conditions are necessary for the development of new diagnostic and therapeutic tools. In a study, it is preferable to employ only a small number of experimental animals, particularly when there are concerns over budgets and over the welfare of the animals. Furthermore, it is necessary to implement minimally invasive in vivo imaging technologies in order continuously to retain a smaller number of animals for experimentation.

Micro-computed tomography (micro-CT) produces high-resolution morphologic images that have been proved to be particularly useful for imaging of small animals [1–3]. It provides spatial information that allows a morphologic correlation of functional data (bone metabolism, blood circulation, metastatic cancer, etc.) with that of other modalities such as optical imaging, positron emission tomography, and magnetic resonance imaging [4–6]. However, several challenges exist for the scaling of clinical images from a mouse to a human adult, because the volume of a mammal’s internal organs is proportional to its body weight. For example, a micro-CT scanner requires voxel sizes of the order of a few micrometers for an adult mouse. The smaller voxels require a significantly higher micro-CT dose for obtaining a high-quality image.

Studies show that the radiation dose during a micro-CT examination may produce adverse effects such as decelerated growth, delayed maturity, or a combination of both morphologic artifacts. The consideration of X-ray doses in small-animal research is different from that in other clinical radiology procedures. One of the main concerns is the prevention of any interference from the effects of radiation in studies that employ the use of CT scans on small animals. Considerations on the long-term radiation effects and the potential for delayed radiation effects are also essential. These factors are particularly important when the images obtained from the mice are used for comparisons of structural changes such as metabolic and cardiac functions. Furthermore, radiation-induced tumor development and radiation-associated tumor growth inhibition must be considered in oncologic studies [7]. The radiation exposure from each scan is not considered to be a limiting factor for long-term studies that use in vivo micro-CT [8].

Because micro-CT imaging requires repetitive examinations in long-term studies of small animals, it is favorable to use only a small number of animals. Because the animals can be examined at different periods of time, this differs from the approaches used in conventional studies that do not require imaging. In these conventional studies, the animals would have to be killed for the correlations between the experiments that use morphologic information to be established. Using radiation dose measurements, Cavanaugh et al. reported that micro-CT scans gave an average dose of 120 mGy, and that radiation damage to the lungs was unlikely. However, they recommended that the frequency of repeated in vivo micro-CT scans must not fall below a weekly one unless an extremely short-term study is performed [9].

There are some papers about the radiation dose for micro-CT scans of experimental animals [10, 11]. However, the detailed relationships between the radiation doses and their subsequent biological effects have never been evaluated in the majority of cases. Thus, we measured the radiation dosage and its corresponding biological effects in ICR mice using cone-beam micro-CT scans.

## Materials and methods

### Micro-CT

Micro-CT imaging was performed with a commercially available micro-CT unit (R_mCT2, RIGAKU, Japan). There are three characteristics of this micro-CT that reduce the effects of X-ray exposure for each scan: (1) The micro-CT scan employed an X-ray flat panel detector that enables a high-speed acquisition of data to obtain low-noise images. During the CT data collection process, a continual scan rather than a stepwise scan is used to reduce the scanning time (17 s) and the X-ray exposure. (2) Reduction of CT noise was accomplished by applying a mean filter to the three-dimensional (3-D) reconstructed data. (3) The X-ray window comprises Be (thickness = 0.15 mm), and the filters comprise Cu (thickness = 0.06 mm) and Al (thickness = 0.5 mm), which cut off the low-energy X-ray domain.

The X-ray focal size was 5 μm, the X-ray tube voltage was set as 90 kV, and the tube current was set to 0.2 mA (Micro focus X-ray source L10101, Hamamatu, Japan). The X-ray detector comprised a CsI scintillator with an amorphous silicon flat panel detector that had an area of 13 cm × 13 cm and a 14-bit resolution of 127 μm per pixel. A computer-controlled object stage was set in the free space of the rotating center in the CT bore. The object stage does not move when the experimental animal is exposed to the X-rays. The field of view was 73 mm × 73 mm. The distance between the rotating center and the X-ray focus was 154 mm, and the distance between the rotating center and the flat-panel surface was 111 mm.

The micro-CT produces high-resolution images with 3-D tomographic data, where a microscopic resolution with a voxel size of less than 148 μm^3^ (148 μm per pixel and slice) is obtained by taking of 514 2-D projections around the sample. The X-ray source produces a cone-shaped beam, which is projected on the specimen. The flat-panel detector senses the modulated X-ray flux density through the specimen. The 514 projections were combined with software that utilizes Feldkamp-type reconstruction with a filtered back-projection algorithm and a cancelation of artifacts. In general, this method is used for the reconstruction of cone-beam CT.

Because the length of a mouse body exceeds 73 mm, two adjacent scans were required for capturing an image of the entire body (whole-body scan mode). In this procedure, the object stage automatically moves to an adjacent position between the first scan and the second scan.

### Dose measurements

**Fig. 1:**
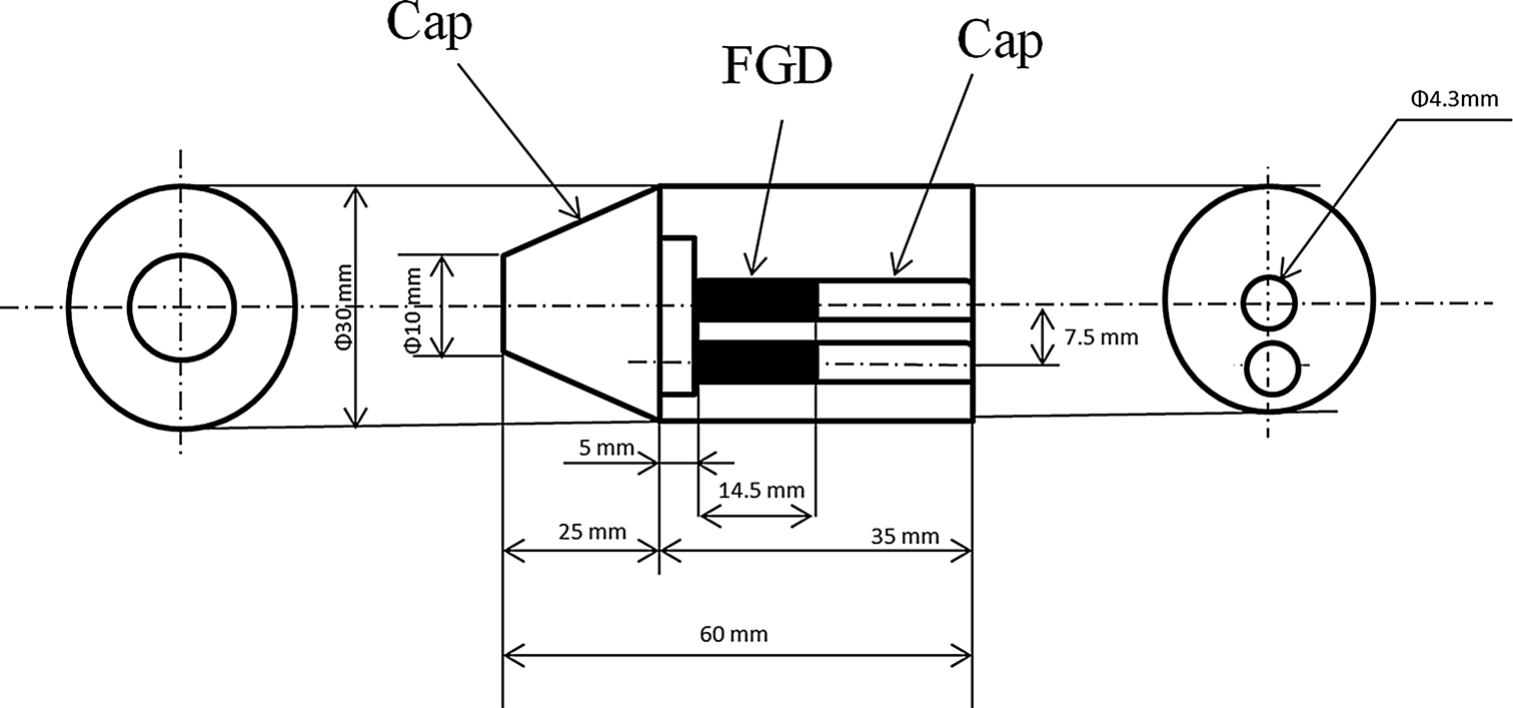
PMMA phantom with inserted fluorescent glass dosimeters (FGDs). The size of the phantom is similar to that of the trunk of the mouse. The two FDGs were exposed simultaneously

The absorbed dose was measured in a circular shaped (diameter: 30 mm and length: 60 mm) polymethylmethacrylate (Fig. 1) phantom, which is designed to mimic a mouse with use of fluorescent glass dosimeters (FGD: GD-352M, Asahi Technoglass, Japan) [12]. The phantom design was based on the micro-CT imaging data of the mice. A fully automated readout system that employs pulse UV-laser excitation (FGD-200, Asahi Technoglass, Japan) was used. The dosimeter rods were positioned in the central axis of the phantom and at an offset position of 7.5 mm from the central axis of the phantom. The phantom was fixed onto the object stage with small pieces of Styrofoam and surgical tape. Prior to the dose measurement, the alignment of the phantom axis with the CT rotational center was checked in a 2-D fluoroscopy mode without the dosimeter. The measurements were obtained simultaneously at both positions with 15 dosimeter rods in each position.

The dosimeters were calibrated with use of an ion chamber [C-110 (0.6 ml), APPLIED ENGINEERING INC., Japan] under an X-ray field. We used the X-ray system (KXO-15E, TOSHIBA, Japan) with a tube voltage of 90 kV, a tube current–time product of 100 mAs, and a source-to-field distance (SFD) of 100 cm. The X-ray filters were Cu (thickness = 0.06 mm) and Al (thickness = 0.5 mm).

### Biological effects of radiation on whole-body CT-scanned ICR mice

All animal experiments were approved by the Institutional Animal Care and Use Committee of Experimental Animals at the National Institute of Radiological Sciences. Six-week-old ICR mice (male: 32, and female: 32) were obtained from Clea Japan and acclimatized for 1 week. The mice were housed at 23 ± 2 °C and a 50 ± 10 % humidity with exposure to a 12 h light–dark cycle and a standard laboratory diet.

**Fig. 2:**
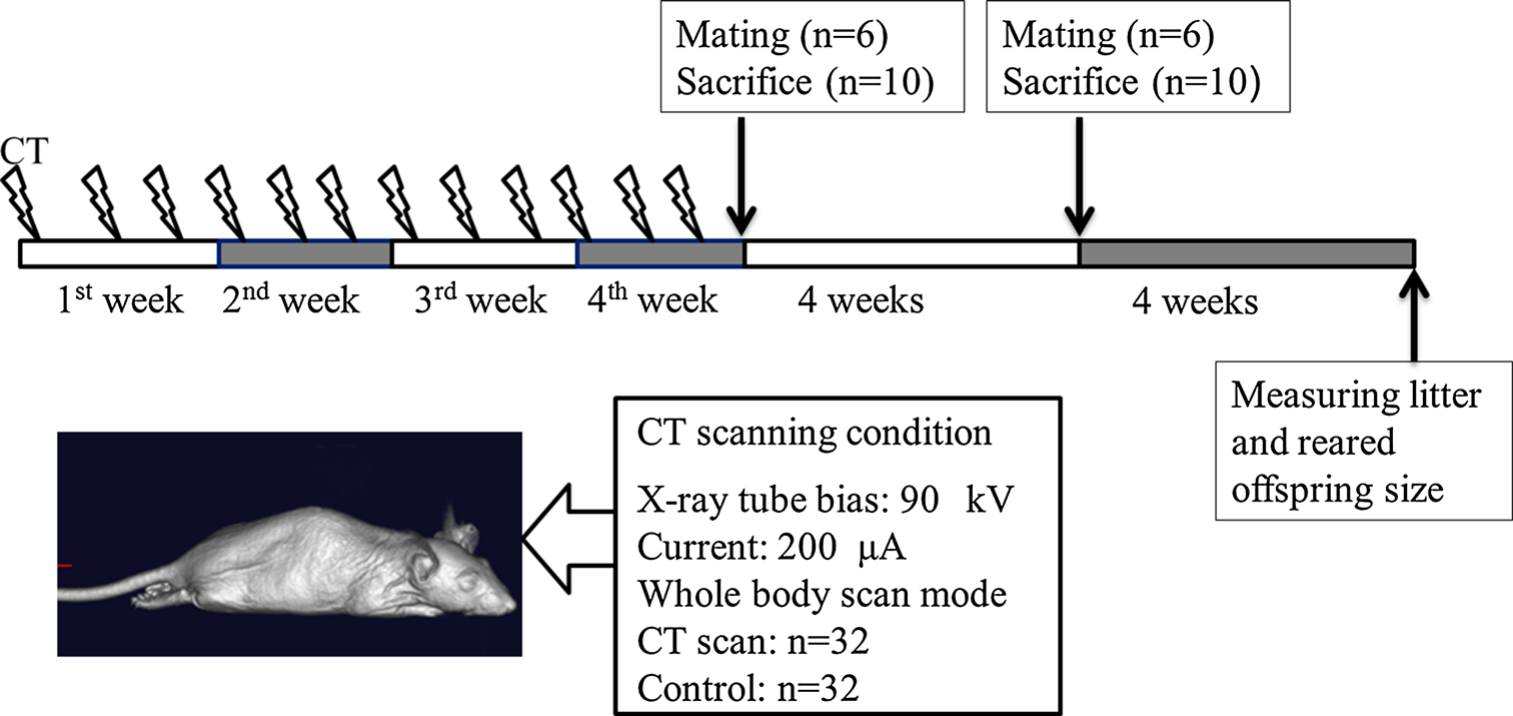
Long-term micro-CT experimental protocol. In vivo long-term whole-body CT scans were performed 3 days per week over a period of 4 weeks

In vivo long-term whole-body CT scans were performed for 3 days per week over of 4 weeks (Fig. 2). The mice were anesthetized with isoflurane gas during the scanning process. Each experimental period lasted for of 8 weeks, the mice were subjected to a long-term CT scan for of 4 weeks and a waiting period of 4 weeks after the last scan. Ten scanned mice and a control group comprising five males and females were immediately sacrificed after this experimental stage for obtaining organ weight measurements and histopathologic observations. Furthermore, three pairs of mice were mated for establishing the litter amounts and sizes of the reared offspring. All of the mated mice were eventually sacrificed after a period of 8 weeks from the last CT scan had passed. The control group and the group of CT-scanned mice were weighed throughout the course of the experiment. The data for the body weight were analyzed with use of two-way repeated-measures ANOVA and Student’s *t* test. The litter amounts and the reared offspring sizes were evaluated by Student’s *t* test.

The animals were anesthetized with an intraperitoneal injection of sodium thiopental prior to necropsy. The brain, thymus, lungs, heart, liver, adrenal glands, kidneys, spleen, testes, epididymides, ovaries, and uterus were then weighed for all the animals. The relative organ weights were calculated on the basis of the overall body weights and evaluated by Student’s *t* test.

Histopathologic examinations were performed on the brain, thymus, lungs, heart, liver, adrenal glands, kidneys, spleen, testes, epididymides, ovaries, uterus, skin, and testes. Hematoxylin and eosin stained specimens were prepared according to standard procedures [13] and examined with a microscope.

Hematology tests were also conducted after the 4-week and the 8-week period for determination of the red blood cell count (RBC), leukocyte count (WBC), and the other 28 blood components (Tables 3, 4, 5, 6). The hematology data were also evaluated by Student’s *t* test.

## Results

In the CT scans, the average measured X-ray dose in the phantom amounted to 16.19 ± 0.808 mGy at the center and 16.24 ± 0.810 mGy at an offset position of 7.5 mm. The highest and lowest doses at the center of the phantom were 16.99 and 15.31 mGy, respectively. The highest and lowest doses at an offset position of 7.5 mm from the center of the phantom were 16.79 and 15.40 mGy, respectively. The total average dose at the center of the phantom after the 4-week long scanning period was 194.28 mGy.

**Fig. 3:**
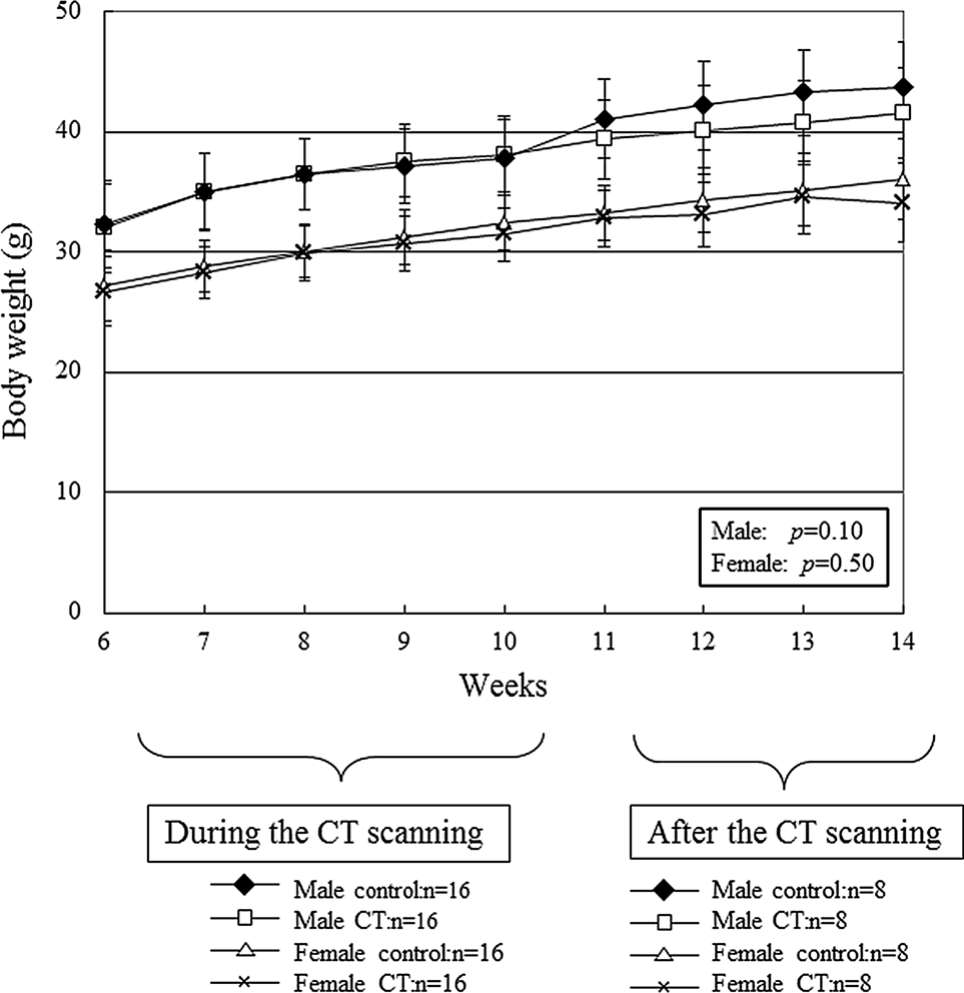
Weight gain curves of the control group and the CT-scanned mice (male: *p* = 0.10, female: *p* = 0.50). No significant differences were found between the mean values

Both the control group and the group of CT-scanned mice showed normal gains in body weight (Fig. 3). No significant differences were observed in the weight gain curves of both groups (*p* = 0.10 for males, *p* = 0.50 for females). The mice were considered to be healthy because they did not exhibit any hair loss or skin erosions throughout the experiment.

The organ weight measurements and the *p* values from Student’s *t* test are shown in Tables 1 and 2. These values also do not reveal any significant differences between the two groups.

**Table 1:** Relative organ weights of the long-term CT-scanned samples after a period of 4 weeks

	Control	After the longitudinal CT (4 weeks)	*p*
(a) Male (*n* = 5)			
Brain (%)	1.17 ± 0.04	1.17 ± 0.07	0.36
Thymus (%)	0.10 ± 0.02	0.08 ± 0.03	0.28
Lung (%)	0.56 ± 0.02	0.51 ± 0.02	0.06
Heart (%)	0.42 ± 0.02	0.43 ± 0.03	0.16
Liver (%)	5.74 ± 0.54	5.82 ± 0.54	0.46
Adrenal glands (%)	0.01 ± 0.00	0.01 ± 0.00	0.39
Kidneys (%)	1.80 ± 0.14	1.81 ± 0.14	0.30
Spleen (%)	0.32 ± 0.08	0.29 ± 0.07	0.18
Testes (%)	0.72 ± 0.05	0.75 ± 0.09	0.38
Epididymides (%)	0.29 ± 0.02	0.26 ± 0.03	0.17
Ovaries (%)	–	–	–
Uterus (%)	–	–	–
(b) Female (*n* = 5)			
Brain (%)	1.38 ± 0.06	1.40 ± 0.07	0.46
Thymus (%)	0.16 ± 0.03	0.23 ± 0.04	0.10
Lung (%)	0.55 ± 0.05	0.54 ± 0.01	0.08
Heart (%)	0.41 ± 0.02	0.40 ± 0.03	0.13
Liver (%)	4.78 ± 0.27	6.32 ± 4.34	0.06
Adrenal glands (%)	0.02 ± 0.00	0.02 ± 0.00	0.43
Kidneys (%)	1.22 ± 0.10	1.21 ± 0.06	0.36
Spleen (%)	0.40 ± 0.03	0.36 ± 0.07	0.07
Testes (%)	–	–	–
Epididymides (%)	–	–	–
Ovaries (%)	0.04 ± 0.01	0.04 ± 0.01	0.41
Uterus (%)	0.41 ± 0.13	0.37 ± 0.05	0.17

There were no significant differences between the mean values

**Table 2:** Relative organ weights of the CT-scanned samples after a period of 8 weeks

	Control	4 weeks after the last scan (8 weeks)	*p*
(a) Male (*n* = 5)			
Brain (%)	1.05 ± 0.02	1.04 ± 0.06	0.36
Thymus (%)	0.10 ± 0.03	0.08 ± 0.02	0.28
Lung (%)	0.49 ± 0.06	0.46 ± 0.02	0.06
Heart (%)	0.44 ± 0.04	0.46 ± 0.02	0.16
Liver (%)	5.93 ± 0.26	5.57 ± 0.44	0.46
Adrenal glands (%)	0.01 ± 0.00	0.01 ± 0.00	0.39
Kidneys (%)	1.79 ± 0.15	1.60 ± 0.12	0.30
Spleen (%)	0.28 ± 0.07	0.29 ± 0.09	0.18
Testes (%)	0.65 ± 0.11	0.65 ± 0.05	0.38
Epididymides (%)	0.28 ± 0.02	0.28 ± 0.04	0.18
Ovaries (%)	–	–	–
Uterus (%)	–	–	–
(b) Female (*n* = 5)			
Brain (%)	1.22 ± 0.08	1.16 ± 0.40	0.46
Thymus (%)	0.14 ± 0.04	0.17 ± 0.03	0.10
Lung (%)	0.52 ± 0.03	0.54 ± 0.06	0.08
Heart (%)	0.45 ± 0.03	0.44 ± 0.04	0.13
Liver (%)	4.63 ± 0.36	5.08 ± 0.16	0.06
Adrenal glands (%)	0.03 ± 0.00	0.03 ± 0.00	0.43
Kidneys (%)	1.15 ± 0.05	1.19 ± 0.08	0.35
Spleen (%)	0.37 ± 0.09	0.34 ± 0.07	0.07
Testes (%)	–	–	–
Epididymides (%)	–	–	–
Ovaries (%)	0.06 ± 0.01	0.07 ± 0.01	0.41
Uterus (%)	0.56 ± 0.20	0.45 ± 0.17	0.18

There were no significant differences between the mean values

Hematology test results are shown in Tables 3, 4, 5 and 6, where an analysis of the results revealed no significant differences between the mean values in terms of the CT scan radiation damage.

**Table 3:** Blood components of the long-term CT-scanned samples after a period of 4 weeks

	Control	After the longitudinal CT (4 weeks)	*p*
(a) Male (*n* = 5)			
RBC (10^4^/μL)	1016.6 ± 170.74	1088.8 ± 107.33	0.28
HGB (g/dL)	15.5 ± 3.94	17.1 ± 3.25	0.18
HCT (%)	49.7 ± 9.01	52.4 ± 6.34	0.38
MCV (fL)	48.8 ± 1.56	48.1 ± 1.24	0.17
MCH (pg)	15.1 ± 1.78	15.6 ± 1.72	0.10
MCHC (g/dL)	30.7 ± 2.89	32.5 ± 2.89	0.08
PLT (10^4^/μL)	134.1 ± 15.47	165.6 ± 30.69	0.13
WBC (10^2^/μL)	74.6 ± 38.21	93.7 ± 25.38	0.06
LYMPH (%)	76.2 ± 7.22	75.2 ± 6.02	0.43
NEUT (%)	14.2 ± 4.48	17.8 ± 6.31	0.36
EO (%)	3.8 ± 2.25	2.2 ± 1.35	0.07
BASO (%)	0.08 ± 0.08	0.02 ± 0.04	0.30
MONO (%)	5.7 ± 1.33	4.7 ± 1.40	0.18
(b) Female (*n* = 5)			
RBC (10^4^/μL)	1062.4 ± 129.60	1013.0 ± 28.88	0.06
HGB (g/dL)	17.9 ± 2.74	15.6 ± 0.75	0.16
HCT (%)	53.9 ± 7.02	50.1 ± 2.05	0.46
MCV (fL)	50.7 ± 1.58	49.4 ± 0.79	0.30
MCH (pg)	16.8 ± 1.16	15.4 ± 0.72	0.30
MCHC (g/dL)	33.1 ± 1.52	31.1 ± 1.75	0.19
PLT (10^4^/μL)	143.1 ± 40.66	143.7 ± 22.68	0.10
WBC (10^2^/μL)	93.4 ± 16.10	79.1 ± 11.72	0.16
LYMPH (%)	82.2 ± 8.05	80.8 ± 4.24	0.46
NEUT (%)	10.6 ± 2.97	12.9 ± 3.97	0.39
EO (%)	3.9 ± 4.49	1.9 ± 0.65	0.30
BASO (%)	0.02 ± 0.04	0.02 ± 0.04	0.18
MONO (%)	3.3 ± 1.59	4.4 ± 1.38	0.38

There were no significant differences between the mean values

**Table 4:** Blood plasma components of long-term CT-scanned samples after a period of 4 weeks

	Control	After the longitudinal CT (4 weeks)	*p*
(a) Male (*n* = 5)			
Total protein (g/dL)	5.52 ± 0.13	5.24 ± 0.15	0.35
Albumin (g/dL)	3.00 ± 0.14	2.82 ± 0.26	0.28
A/G	1.20 ± 0.12	1.19 ± 0.24	0.07
BUN (mg/dL)	27.36 ± 1.67	23.80 ± 2.48	0.16
CRE (mg/dL)	0.06 ± 0.01	0.06 ± 0.004	0.46
Total Cholesterol (mg/dL)	130.40 ± 24.03	119.00 ± 21.60	0.39
Total bilirubin (mg/dL)	0.08 ± 0.02	0.12 ± 0.05	0.30
Na (mEq/L)	154.40 ± 1.52	153.80 ± 1.30	0.18
K (mEq/L)	7.70 ± 1.35	7.38 ± 1.61	0.36
Cl (mEq/L)	106.20 ± 2.68	106.20 ± 1.92	0.28
ALP (U/L)	219.20 ± 224.80	224.80 ± 105.43	0.09
ALT (U/L)	35.20 ± 5.54	44.60 ± 9.04	0.16
AST (U/L)	52.00 ± 4.12	57.40 ± 7.83	0.46
LDH (U/L)	1026.40 ± 109.49	963.40 ± 170.28	0.39
γ-GT (U/L)	4.00 ± 0.71	2.80 ± 0.84	0.30
CPK (U/L)	151.60 ± 40.95	245.20 ± 198.64	0.18
TG (mg/dL)	86.80 ± 24.63	53.00 ± 15.25	0.18
(b) Female (*n* = 5)			
Total protein (g/dL)	5.18 ± 0.25	5.04 ± 0.18	0.10
Albumin (g/dL)	3.10 ± 0.12	3.08 ± 0.08	0.08
A/G	1.49 ± 0.05	1.58 ± 0.12	0.13
BUN (mg/dL)	17.02 ± 2.91	17.00 ± 3.26	0.06
CRE (mg/dL)	0.08 ± 0.01	0.08 ± 0.01	0.43
Total cholesterol (mg/dL)	96.20 ± 16.54	97.00 ± 17.72	0.35
Total bilirubin (mg/dL)	0.05 ± 0.02	0.07 ± 0.05	0.07
Na (mEq/L)	150.80 ± 1.30	148.60 ± 0.89	0.38
K (mEq/L)	7.06 ± 1.33	8.04 ± 0.92	0.47
Cl (mEq/L)	109.20 ± 2.49	108.20 ± 2.77	0.13
ALP (U/L)	335.40 ± 93.70	352.00 ± 59.78	0.08
ALT (U/L)	36.80 ± 11.54	28.20 ± 6.06	0.13
AST (U/L)	56.60 ± 13.76	48.00 ± 4.30	0.06
LDH (U/L)	416.40 ± 134.48	227.80 ± 82.90	0.44
γ-GT (U/L)	4.40 ± 1.82	3.80 ± 1.48	0.36
CPK (U/L)	81.40 ± 32.39	46.40 ± 9.89	0.07
TG (mg/dL)	79.60 ± 15.53	95.00 ± 27.01	0.46

There were no significant differences between the mean values

**Table 5:** Blood components of the CT-scanned samples after a period of 8 weeks

	Control	4 weeks after the last scan (8 weeks)	*p*
(a) Male (*n* = 5)			
RBC (10^4^/μL)	1035.80 ± 72.68	1068.80 ± 85.91	0.07
HGB (g/dL)	16.82 ± 1.36	17.08 ± 1.32	0.21
HCT (%)	51.20 ± 3.35	51.94 ± 4.60	0.06
MCV (fL)	49.46 ± 1.29	48.56 ± 0.75	0.14
MCH (pg)	22.24 ± 13.53	16.00 ± 0.25	0.08
MCHC (g/dL)	32.84 ± 0.94	32.92 ± 0.51	0.27
PLT (10^4^/μL)	147.48 ± 14.19	138.96 ± 13.75	0.06
WBC (10^2^/μL)	61.52 ± 18.82	66.44 ± 17.00	0.34
LYMPH (%)	73.38 ± 11.16	78.12 ± 5.62	0.44
NEUT (%)	18.40 ± 8.29	14.70 ± 5.40	0.07
EO (%)	2.40 ± 1.82	2.10 ± 0.91	0.11
BASO (%)	0.06 ± 0.09	0.02 ± 0.04	0.15
MONO (%)	5.76 ± 2.85	5.06 ± 0.64	0.12
(b) Female (*n* = 5)			
RBC (10^4^/μL)	1047.00 ± 76.73	986.75 ± 9.18	0.49
HGB (g/dL)	16.42 ± 1.56	15.73 ± 0.40	0.21
HCT (%)	50.04 ± 3.05	48.45 ± 0.51	0.30
MCV (fL)	47.76 ± 1.30	41.58 ± 15.00	0.26
MCH (pg)	15.68 ± 0.56	45.93 ± 60.25	0.09
MCHC (g/dL)	32.80 ± 1.36	32.45 ± 0.66	0.16
PLT (10^4^/μL)	107.72 ± 25.88	109.63 ± 20.52	0.09
WBC (10^2^/μL)	47.94 ± 15.91	48.75 ± 14.69	0.23
LYMPH (%)	81.42 ± 0.76	80.48 ± 1.33	0.10
NEUT (%)	13.30 ± 1.85	12.25 ± 1.41	0.33
EO (%)	1.78 ± 1.00	3.43 ± 1.88	0.49
BASO (%)	0.01 ± 0.01	0.01 ± 0.01	0.37
MONO (%)	3.50 ± 1.46	3.85 ± 2.33	0.28

There were no significant differences between the mean values

**Table 6:** Blood plasma components of the CT-scanned samples after a period of 8 weeks

	Control	4 weeks after the last scan (8 weeks)	*p*
(a) Male (*n* = 5)			
Total protein (g/dL)	5.38 ± 0.25	5.22 ± 0.31	0.32
Albumin (g/dL)	3.00 ± 0.21	2.80 ± 0.28	0.07
A/G	1.27 ± 0.13	1.16 ± 0.16	0.15
BUN (mg/dL)	29.70 ± 2.25	24.64 ± 4.13	0.13
CRE (mg/dL)	0.07 ± 0.01	0.07 ± 0.01	0.42
Total cholesterol (mg/dL)	112.60 ± 17.94	136.20 ± 28.37	0.29
Total bilirubin (mg/dL)	0.04 ± 0.02	0.04 ± 0.01	0.30
Na (mEq/L)	154.60 ± 0.89	156.00 ± 0.71	0.26
K (mEq/L)	7.80 ± 1.54	7.62 ± 0.80	0.08
Cl (mEq/L)	107.60 ± 3.21	109.20 ± 2.05	0.14
ALP (U/L)	198.80 ± 48.84	180.80 ± 51.66	0.07
ALT (U/L)	49.20 ± 11.52	39.80 ± 8.61	0.23
AST (U/L)	69.80 ± 21.23	50.40 ± 6.80	0.08
LDH (U/L)	1019.60 ± 211.82	668.80 ± 36.87	0.32
γ-GT (U/L)	2.40 ± 0.55	2.40 ± 0.55	0.42
CPK (U/L)	124.60 ± 16.55	115.60 ± 31.73	0.47
TG (mg/dL)	86.00 ± 25.33	111.00 ± 26.02	0.22
(b) Female (*n* = 5)			
Total protein (g/dL)	5.14 ± 0.09	4.98 ± 0.24	0.14
Albumin (g/dL)	3.16 ± 0.13	3.18 ± 0.10	0.07
A/G	1.60 ± 0.14	1.77 ± 0.08	0.23
BUN (mg/dL)	20.86 ± 2.70	19.93 ± 2.23	0.08
CRE (mg/dL)	0.09 ± 0.004	0.09 ± 0.01	0.32
Total cholesterol (mg/dL)	104.60 ± 12.42	75.00 ± 8.04	0.42
Total bilirubin (mg/dL)	0.04 ± 0.001	0.06 ± 0.02	0.47
Na (mEq/L)	152.20 ± 0.84	150.75 ± 1.50	0.22
K (mEq/L)	6.98 ± 0.78	7.23 ± 0.59	0.32
Cl (mEq/L)	110.40 ± 1.52	111.00 ± 2.16	0.07
ALP (U/L)	264.80 ± 34.78	281.25 ± 99.95	0.15
ALT (U/L)	28.60 ± 6.58	33.00 ± 8.83	0.13
AST (U/L)	47.40 ± 5.73	49.50 ± 5.74	0.42
LDH (U/L)	383.80 ± 96.59	309.50 ± 54.93	0.29
γ-GT (U/L)	2.60 ± 0.55	2.00 ± 0.01	0.30
CPK (U/L)	64.40 ± 21.62	41.75 ± 6.75	0.25
TG (mg/dL)	77.80 ± 17.63	77.50 ± 19.02	0.08

There were no significant differences between the mean values

**Fig. 4:**
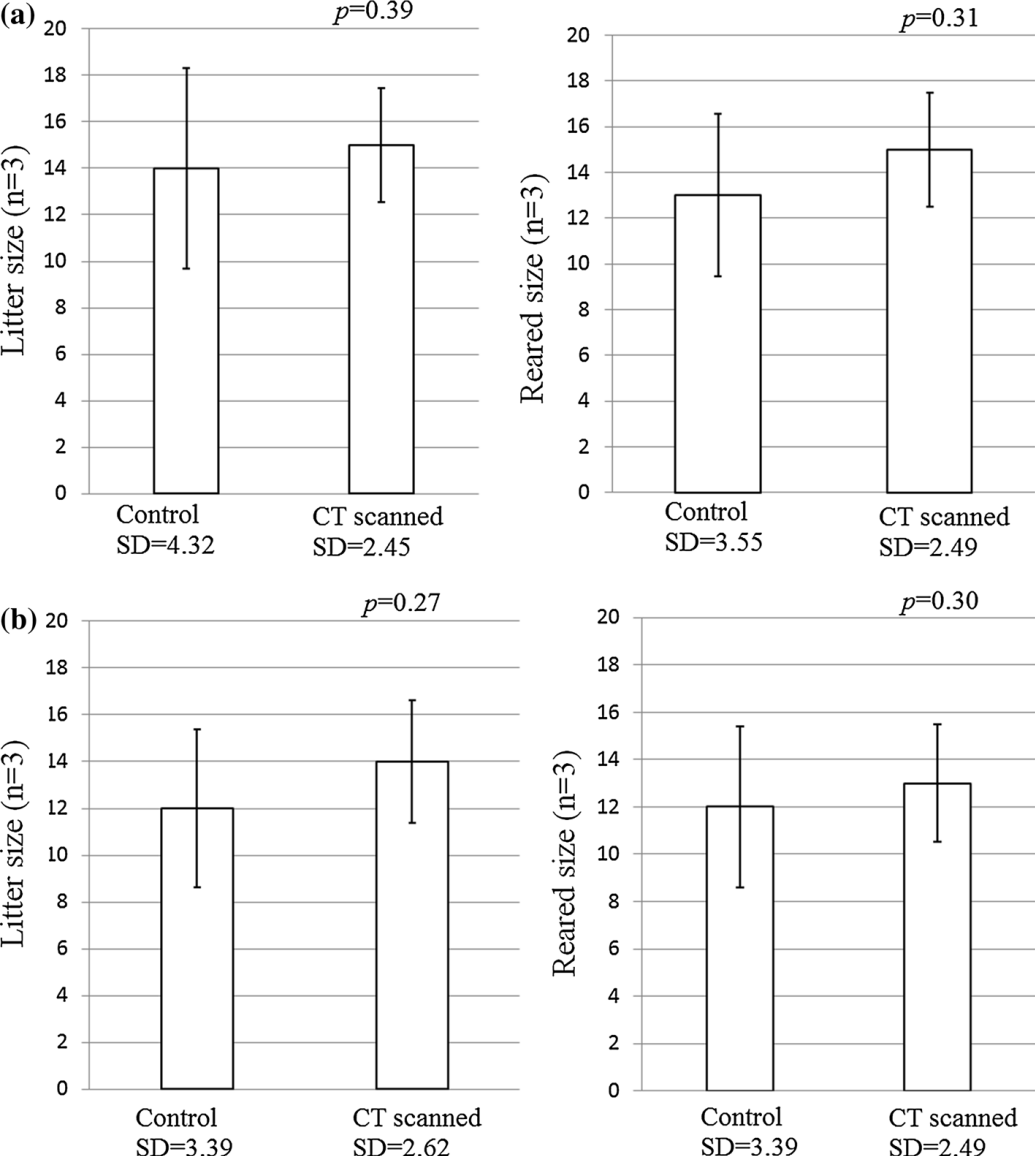
Litter sizes and reared offspring sizes. No significant differences were found between the mean values. **a** Mated after long-term CT (4 weeks, *n* = 3). **b** Mated 4 weeks after the last scan (8 weeks, *n* = 3)

The litter size and the sizes of the reared offspring from the mated mice after 4 weeks and after 8 weeks are shown in Fig. 4. No changes were detected between the control group and the group of CT-scanned mice in terms of the litter amounts and the sizes of the reared offspring after a 4-week period of long-term CT scans (litter size: *p* = 0.39, reared off spring size: *p* = 0.30) and after an additional period of 4 weeks after the last scan (8 weeks) (litter size: *p* = 0.27, reared offspring size: *p* = 0.30).

**Fig. 5:**
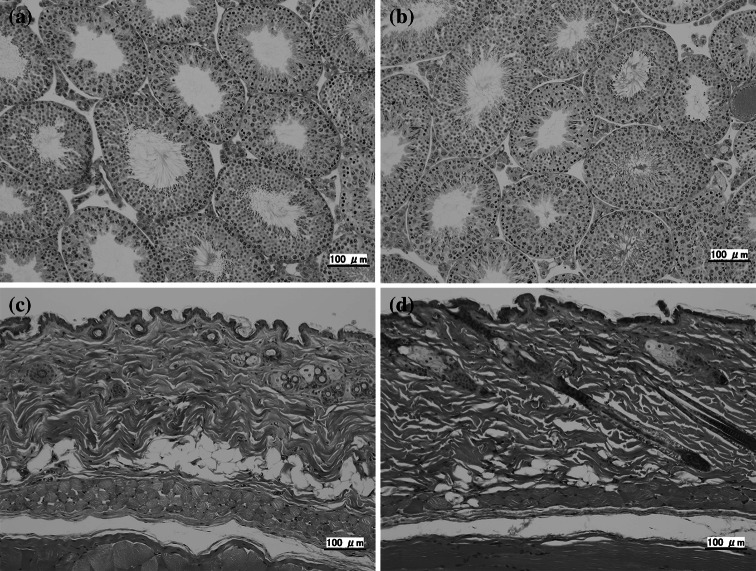
Histopathologic observations (hematoxylin and eosin stained specimens). All samples were examined by microscope. **a** Control (testis). **b** Four weeks after the last scan (8 weeks: testis). No abnormal vasodilation or edematous tissue was observed. **c** Control (skin). **d** Four weeks after the last scan (8 weeks: skin). No damaged keratinocytes or desquamation was observed

Histopathologic observations of the skin and testes are shown in Fig. 5. Any histopathologic feature of radiation-damaged keratinocytes via apoptosis and vasodilation in the tissues would be an important discovery. However, there were no damaged keratinocytes or desquamation in the skin samples, and abnormal vasodilation and edematous tissue were not observed in any of the testes samples. Furthermore, there were no abnormal histopathologic findings in the other ten organs after the 4- and 8-week experimental periods.

## Discussion

In this study, long-term in vivo whole-body micro-CT scans with measured X-ray doses were performed on ICR mice for 4 weeks with R-mCT2. A scanning frequency of three scans per week is higher than that necessary for conventional studies that employ only approximately one or two scans per week. However, this is designed to represent cases where subjects receive an extreme dose of X-rays during an examination. The total dose at the center of the phantom was measured to be 194.3 mGy over a duration of 4 weeks. However, weight gain curves, organ weights, blood analyses, litter sizes, reared offspring sizes, and histopathologic findings showed that the radiation did not have any significant impact on the mice.

FGD rods were set at the center of the phantom and at an offset position of 7.5 mm from the center of the phantom. It is unlikely that these positional differences affected the resultant dose distribution within the phantom. The deviation of the fluorescent dosimeter sensitivity (16.19 ± 0.808 mGy at the center, 16.24 ± 0.810 mGy at an offset position of 7.5 mm) is too large to allow an accurate measurement of the distributed dose within the phantom. A substantially smaller dosimeter and a more precise measurement technique must be implemented for either measuring or estimating the specific dose to each of the organs in the mice.

Deviations in the appearance of chromosomal aberrations for mammals were also observed at higher doses of 250–300 mGy in other studies [14, 15]. In this study, the total average dose was 194.3 mGy for the CT scans, which means that this is unlikely to cause any radiation damage to mice. Taschereau et al. reported that the average whole-body dose for a long-term micro-CT examination was approximately 160 mGy [8]. In studies on lethal radiation doses in mice, the LD_50/28_ was 26,800 mGy for a single dose, but increased to 35,300 and 57,500 mGy for 2 and 10 fractions, respectively [16]. Rodents that have been exposed to sublethal doses of radiation have the ability to repair the effects from these doses within a few hours. This is similar to fractionated radiation treatment, in which long-term micro-CT studies may include administration of repetitive radiation doses with comparatively low side effects. It has been shown that repetitive radiation with micro-CT scans may have adverse effects on tumor growth in small-animal studies. However, the reported therapeutic radiation dose values were of the order of a few Gy [17], which rules out any potential therapeutic effects.

Several histopathologic patterns are produced by ionizing radiation in the tissues. The most commonly observed effect of irradiation is atrophy, which may occur in the skin lining, the glands, and the alimentary, respiratory, and urinary tracts. Necrosis occurs in the majority of the epithelial and parenchymal cells during the acute phase of radiation injury, and metaplasia can also be observed in squamous epithelial metaplasia. Fibrosis is another commonly observed effect of irradiation. Another dominant effect of acute damage is a reduction in the microvascular network, which ultimately results in ischemia. However, none of these histologic findings were confirmed in any of our samples in this study. This is because the dose from the CT scan was 194.3 mGy, which is unlikely to cause any radiation damage to mice. However, the number of CT scans, the X-ray doses, and the ensuing effects of radiation should be carefully considered, particularly when the absorbed dose increases above the threshold dose (250–300 mGy).

## Conclusion

Micro-CT (i.e., R_mCT2) scans generally give very low doses of radiation to a subject. For example, frequent micro-CT scans for a period of 4 weeks only give an average dose of 194.3 mGy. It is unlikely that an extremely low dose can cause any acute radiation damage to mice. We did not find any indication that the performance of long-term CT scans for a duration of 4 weeks would affect the resulting body weight gain, organ weight measurements, hematology test results, litter sizes, reared offspring sizes, or histopathologic findings. However, the number of CT scans, the radiation dose from the X-rays, and the ensuing effects of radiation should be considered carefully, particularly when a different experimental protocol is used.

## References

[1] Cody DD, Nelson CL, Bradley WM, Wislez M, Juroske D, Price RE, Zhou X, Bekele BN, Kurie JM (2005). Murine lung tumor measurement using respiratory-gated micro-computed tomography. Invest Radiol.

[2] Ford NL, Graham KC, Groom AC, MacDonald IC, Chambers AF, Holdsworth DW (2006). Time-course characterization of the computed tomography contrast enhancement of an iodinated blood-pool contrast agent in mice using a volumetric Flat-Panel equipped computed tomography scanner. Invest Radiol.

[3] Udagawa A, Sato S, Hasuike A, Kishida M, Arai Y, Ito K (2013). Micro-CT observation of angiogenesis in bone regeneration. Clin Oral Implants Res.

[4] Hargreaves RJ (2009). The role of molecular imaging in drug discovery and development. Clin Pharmacol Ther.

[5] Rodt T, Luepke M, Boehm C, Hueper K, Halter R, Glage S, Hoy L, Wacker F, Borlak J, von Falck C (2012). Combined micro-PET/micro-CT imaging of lung tumours in SPC-raf and SPC-myc transgenic mice. PLoS One.

[6] Rodt T, von Falck C, Halter R, Ringe K, Shin HO, Galanski M, Borlak J (2009). In vivo microCT quantification of lung tumor growth in SPC-raf transgenic mice. Front Biosci.

[7] Stone HB, Coeman CN, Anscher MS, McBride WH (2003). Effects of radiation on normal tissue: consequences and mechanisms. Lancet.

[8] Taschereau R, Chow PL, Chatziioannou AF (2006). Monte Carlo simulations of dose from microCT imaging procedures in a realistic mouse phantom. Med Phys.

[9] Cavanaugh D, Johnson EJ, Price RE, Kurie J, Travis EL, Cody DD (2004). In vivo respiratory-gated micro-CT imaging in small-animal oncology models. Mol Imaging Biol.

[10] Bartling SH, Stiller W, Semmler W, Kiessling F (2007). Small animal computed tomography Imaging. Curr Med Imaging Rev.

[11] De Clerck KM, Weiler H, Van Dyck D, Vanhoutte G, Terpstra P, Postnov A (2004). High-resolution X-ray microtomography for the detection of lung tumors in living mice. Neoplasia.

[12] Hoshi Y, Nomura T, Oda T, Iwasaki T, Fujita K, Ishikawa T, Kato A, Ikegami T, Sakai K, Tanooka H, Yamada T (2000). Application of a newly developed photoluminescence glass dosimeter for measuring the absorbed dose in individual mice exposed to low-dose rate 137Cs γ-rays. Radiat Res.

[13] Berezovsky ME (1978). Method for staining semi-thin sections with hematoxylin–eosin. Ark Patol.

[14] Luchnik NV, Sevankaev AV (1976). Radiation-induced chromosomal aberrations in human lymphocytes. I. Dependence on the dose of gamma-rays and an anomaly at low doses. Mutat Res.

[15] Takahashi E, Hirai M, Tobari E, Utsugi T, Nakai S (1982). Radiation-induced chromosome aberrations in lymphocytes from man and crab-eating monkey: the dose-response relationships of low doses. Mutat Res.

[16] Phillips TL, Ross G (1974). Time–dose relationships in the mouse esophagus. Radiology.

[17] Rodt T, Luepke M, Boehm C, von Falck C, Stamm G, Borlak J, Seifert H, Galanski M (2011). Phantom and cadaver measurements of dose and dose distribution in micro-CT of the chest in mice. Acta Radiol.

